# Alterations of Gut Microbiota in Type 2 Diabetes Individuals and the Confounding Effect of Antidiabetic Agents

**DOI:** 10.1155/2020/7253978

**Published:** 2020-09-28

**Authors:** Babiker Saad Almugadam, Yinhui Liu, Shen-min Chen, Chun-hao Wang, Chen-yi Shao, Bao-wei Ren, Li Tang

**Affiliations:** ^1^Department of Microecology, College of Basic Medical Sciences, Dalian Medical University, Dalian, Liaoning, China; ^2^Department of Microbiology, Faculty of Medical Laboratory Sciences, University of El Imam El Mahdi, Kosti, White Nile State, Sudan

## Abstract

Type 2 diabetes is a leading cause of morbidity and a common risk of several disorders. Identifying the microbial ecology changes is essential for disease prediction, therapy, and prevention. Thus, our study is aimed at investigating the intestinal microbiota among healthy and type 2 diabetes individuals and exploring the effect of antidiabetic agents on gut bacterial flora. 24 type 2 diabetes (metformin, glimepiride, and nontherapeutic subgroups; *N* = 8) and 24 healthy control subjects were enrolled in this study, and intestinal bacterial microbiota was investigated by analyzing V3-V4 regions of 16S rRNA gene sequence. Numerous alterations were observed in the gut microbial community of diabetic individuals. These changes were characterized by a significant lowered abundance of *Faecalibacterium*, *Fusobacterium*, *Dialister*, and *Elusimicrobium* in the nontherapeutic subgroup compared to the healthy control group. Likewise, correlation analysis showed a substantial decline in gut microbiota richness and diversity with the duration of illness. Furthermore, antidiabetic agents restored to some extent the richness and diversity of gut microbiota and improved the abundance of many beneficial bacteria with a significant increase of *Methanobrevibacter* in the metformin subcategory compared to the nontherapeutic subgroup. In return, they decreased the abundance of some opportunistic pathogens. The findings of this study have added a novel understanding about the pathogenesis of the disease and the mechanisms underlying antidiabetic therapy, which are of potential interest for therapeutic lines and further studies.

## 1. Introduction

Gut microbiota are miscellaneous groups of microorganisms that inhabit the gastrointestinal tract (GIT) of both humans and animals. It has coexisted with the body in a symbiotic relationship, with significant metabolic and regulatory functions. The intestinal microbiota are modulated by dietary and cultural habits, age, exercise, ethnicity, and genetics, which are distinctive and extremely variable among individuals [1, 2].

Type 2 diabetes mellitus (T2DM) belongs to a cluster of broadly distributed chronic disorders that result from the disruption of sugar metabolism and homeostasis. It has complicated mechanisms and multiple factors implicated [3, 4]. Globally, T2DM is a widely distributed disease and a leading cause of morbidity, with approximately more than 500 million new cases in 2018 [4, 5]. Throughout 2010 to 2030, the expected rise is 20% and 69% in developed and developing countries, respectively [3]. Notably, T2DM is increasing the risk of infections, eye disorders, and kidney illnesses [3]. Furthermore, the prediction of diabetes as the seventh cause of mortality by 2030 introduces the urgent need for efficient preventive and therapeutic approaches, which include maintaining a healthy ecosystem, lifestyle, and feeding habits [6]. Recently, increasing evidence suggests that the intestinal flora has a significant effect in the development of metabolic disorders, which is attributed to dysbiosis of microbial communities and metabolites. Many dietary components are metabolized by intestinal commensal flora to yield a potent metabolic, body physiology, and immune regulators. Indeed, short-chain fatty acids (SCFAs) are common products of microbial metabolism from nondigestible carbohydrates. Recently, numerous studies have shown that SCFAs reduce body weight and insulin resistance, thereby suppressing appetite and lipolysis, increasing energy expenditure and oxidative metabolism, and enhancing insulin sensitivity and production [7–9]. Dysbiosis of gut microbiota disturbs the microbial ecosystem and predisposes to physiological alterations and diseases [10]. Insulin resistance does not exclusively result from overweight but also involves a complex interplay of multiple factors such as the gut ecosystem and immune response.

Taken together, the immunologic, metabolic, and regulatory potential of intestinal microflora explains it is significance in health and diseases. Understanding the microbial ecology will help us to recognize the vital ecosystem and provide the essential information for disease prediction and designing specific strategies to modulate gut microbial flora for preventive and therapeutic purposes. In consequence, to clarify the change in the intestinal flora and it is potential in the pathogenesis of T2DM, many studies are necessary. Thus, our study is aimed at investigating the gut microbiota among healthy and type 2 diabetes individuals and exploring the effect of antidiabetic agents on gut microbial flora.

## 2. Materials and Methods

### 2.1. Study Design, Area, and Duration

This was a case-control study carried out at Namuzaji Center of National Health Insurance Fund, Kosti, White Nile State, Sudan. Throughout January to March 2019, an assembly of Sudanese healthy and type 2 diabetes individuals were randomly invited and enrolled as study contributors. The research protocol had been approved by the Ethics Review Committee of the Namuzaji Center of National Health Insurance Fund in 2019 and applied the Declaration of Helsinki for the research regarding the human subjects. A researcher invited the participants and explained the protocol in detailed information, and written informed consent obtained from everyone.

### 2.2. Study Subjects and Data Collection

The study population consists of healthy control (HC) and T2DM individuals diagnosed according to the strategies of the American Diabetes Association. Participant selection was based on the set of targeted criteria, and the main eligibility criterion was aged > 18 years regardless of gender and ethnic group. Based on the treatment protocol, the diabetic group was further categorized into three clusters (*N* = 8): metformin (MET), glimepiride (GLIM), and nontherapeutic (NT) subgroups. The eligible subject in T2DM subgroups was at least ≥one month under the daily dose of MET 500 mg (3 times per day), once a dose of GLIM 2 mg, or NT management policy. The participant's exclusion criteria were aged ≤ 18 years, history of acute or chronic illnesses, antimicrobial use at least before 4 weeks, cancer or autoimmune therapy, previous history of GIT surgery or symptomatic disease, pregnancy and breastfeeding, alcohol consumption, and smoking. People who suffered from GIT parasitosis, abdominal disturbance, diarrhea, dysentery or diabetes complications have also been excluded. The participant's information including gender, age, marital status, residence, education level, occupation, height, and weight was gathered using a constructed questionnaire; and body mass index (BMI) also calculated. All the recruited participants had no apparent indication of illness (excluding T2DM for the study cases) as confirmed by clinical examination, past medical history, and laboratory investigations.

### 2.3. Sample Collection and Laboratory Analysis

Early morning, fresh fecal sample was collected from every individual using sterile stool container and immediately divided into two parts. Afterward, one part was used for the investigation of GIT parasites, whereas the other part was kept at -20°C prior the DNA extraction [11]. The lack of asymptomatic intestinal parasitic infection among groups and T2DM in HC subjects was confirmed by using direct saline, formal-ether concentration technique and modified Ziehl–Neelsen (ZN) method; and oral glucose tolerance test (OGTT), respectively. These investigations were performed as described previously [12]. About 2-5 ml of fasting venous blood specimen was collected from each person in fluoride oxalate container in the morning, along with 1-2 ml of fasting venous blood sample from every individual of the T2DM group in EDTA container and 2-3 ml of venous blood sample from every person of the HC group in fluoride oxalate container at 2 h after the standard glucose dose as described in OGTT [12]. Fluoride oxalate samples were centrifuged at 3000 rpm for 5 minutes to obtain plasma that was processed immediately for fasting blood glucose (FBG) or 2 h blood glucose using a semiautomated biochemistry analyzer (Mindray BA-88A) and BioSystems glucose kit. EDTA samples were subjected to measurement of hemoglobin A1c (HbA1c) level by Boditech i-CHROMA™ instrument and kit based on company protocol.

### 2.4. DNA Extraction and Bacterial 16S rRNA Gene Sequencing

Bacterial DNA was extracted from 200 mg of every fecal specimen by E.Z.N.A.® Stool DNA Kit (OMEGA Bio-tek, Inc) based on company guidelines. NanoDrop 2000 (Thermo Fisher Scientific, USA) had used to assess the quality (Purity and concentration) of the extracted DNA. Subsequently, all the extracted DNA specimens were stored at −80°C until the next step. For DNA sequencing, V3-V4 regions of 16S rRNA gene sequences were amplified by using specific primers with barcode (341F: 5-CCTAYGGGRBGCASCAG-3 and 806R: 5-GGACTACNNGGGTATCTAAT-3). All PCR reactions were carried out in 30 *μ*l reaction volume with 15 *μ*l of Phusion® High-Fidelity PCR Master Mix (New England Biolabs), 1 *μ*l of each primer, 1 *μ*l of DNA, and 12 *μ*l of ddH_2_O. PCR conditions were done with an initial denaturation at 98°C for 1 min; 30 cycles of denaturation at 98°C for 10 s, annealing at 50°C for 30 s, and elongation at 72°C for 30 s; and a final extension at 72°C for 5 minutes. The amplified 16S rRNA genes were purified by GeneJET™ Gel Extraction Kit (Thermo Scientific) and sequencing library constructed using Ion Plus Fragment Library Kit 48 rxns (Thermo Scientific) following the manufacturer's recommendations. Afterward, the constructed library had subjected to Qubit quantification and library testing (Qubit@ 2.0 Fluorometer, Thermo Scientific), and then sequenced using the Ion S5™ XL platform.

### 2.5. Sequence Quality Control, Operational Taxonomic Units Clustering, and Species Annotation

Quality filtering on the raw reads was performed under specific filtering conditions [13], and Chimera sequences had also identified and removed to obtain high-quality clean reads [14, 15]. Sequence analyses were performed using Uparse software (Uparse v7.0.1001) [16]. Sequences with ≥97% similarity were assigned to the same Operational Taxonomic Units (OTUs). Next, the representative sequence for each OTU was annotated and characterized by classification levels [17, 18]. Multiple sequence alignment was also performed to obtain the phylogenetic relationship for all OTUs and difference in dominant taxa between groups [19]. Finally, the data were normalized and the subsequent analyses performed.

### 2.6. Data Analysis

Statistical analyses of the study data were conducted using IBM SPSS Statistics for Windows, version 21.0 (IBM Corp., Armonk, NY, USA). Categorical data were presented as number and numerical data as mean. Two independent-samples *t*-test, Mann-Whitney *U* test, one-way ANOVA, and Kruskal-Wallis test assessed the difference in numerical data. Fisher's exact test was involved in categorical data as well. A *P* value of less than 0.05 was considered significant.

Alpha diversity indices (Observed species, Chao1, ACE, Shannon, and Simpson) were analyzed to see the difference in gut microbiota richness (Observed species, Chao1, and ACE) and diversity (Shannon and Simpson). PCoA, NMDS, and beta diversity were assessed with QIIME software (version 1.9.1) and presented by R software (version 2.15.3). Biomarkers were determined by a LEfSe analysis based on LEfSe software with the default setting of LDA score at 4. Furthermore, the Anosim test and Spearman correlation analysis were also involved in the analysis of difference between groups and the relationship of some variables with relative abundance taxa, respectively.

## 3. Results

### 3.1. Characteristics of the Study Participants

Forty-eight participants (24 HC and 24 T2DM individuals) were recruited in this study. Between HC and either T2DM individuals or NT subgroup, there was no significant variations in the distribution of the study participants among gender, marital status, residence, education level, and occupation as well as BMI. Likewise, there was no significant difference in FBG and disease duration between T2DM subgroups (MET, GLIM, and NT). The male/female ratio was one in both HC and T2DM groups. The average estimated age was 53.87 in diabetic and 47.12 in HC, *P* = 0.024. Notably, there was a significant variations in HbA1c between T2DM subgroups, *P* = 0.044 (Table 1).

### 3.2. Diversity of Gut Microbial Community

**Figure 1:**
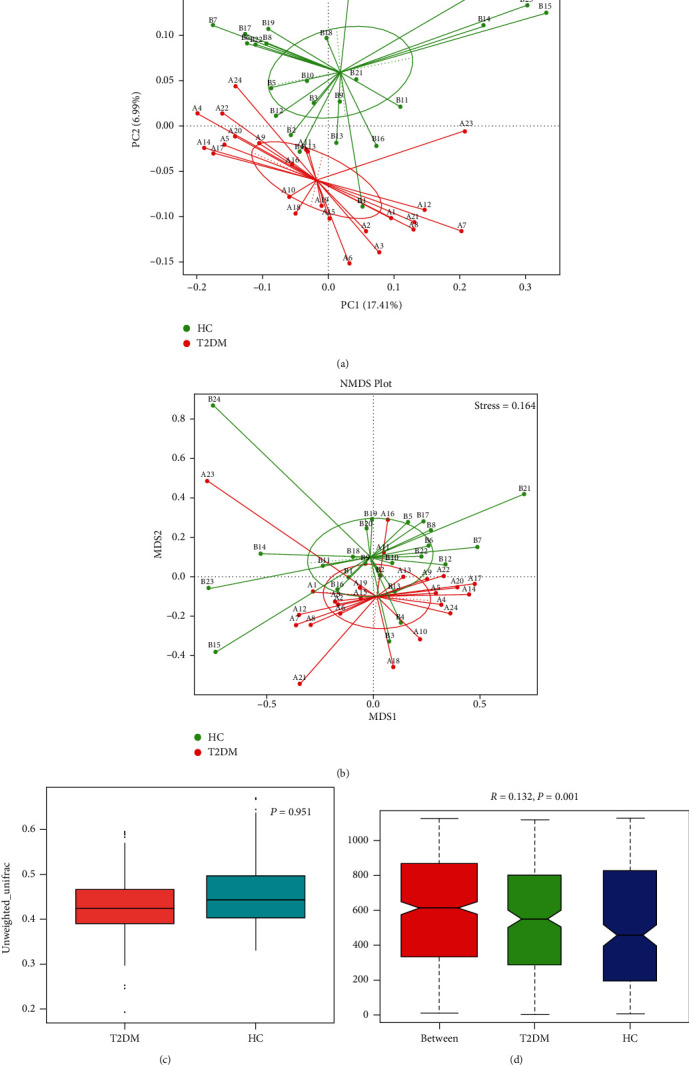
Multisample comparison analysis (beta diversity indices) between the type 2 diabetes mellitus (T2DM) group and healthy control (HC) individuals. PCoA based on (a) unweighted_unifrac, (b) NMDS Plot, and (c) unweighted beta diversity compared the microbial community of the type 2 diabetes group (*n* = 24) with healthy individuals (*n* = 24). (d) Anosim analysis describes the variation between and within the groups. *R* value is between -1 and 1. *R* value greater than zero indicates a significant difference between groups, while less than zero indicates that the difference within the group is greater than between the groups. The reliability of the statistical analysis is represented by probability value (*P* < 0.05 indicates that the statistic is significant). A: label of T2DM samples; B: label of HC samples; *n*: number.

The evaluation of OTUs in terms of diversity indices revealed marked variations between groups. Alpha diversity measures including Shannon and Simpson indices and richness estimators (Chao1, ACE, and Observed species) were nonsignificantly different between the T2DM and HC groups; however, the abundance of gut microbiota was higher in T2DM compared to HC as noticed in Chao1, Observed species, and ACE indices. Likewise, based on Shannon index, bacterial diversity was higher in the HC group compared to T2DM subjects (Table 2). On the other hand, although *β*-diversity based on unweighted unifrac had shown no significant difference between groups, all of the principal coordinate analyses (unweighted unifrac, PCoA) and NMDS Plot, have exhibited a distinct separation between T2DM and HC samples (Figures 1(a)–1(c)). Furthermore, Anosim test found that the study groups (T2DM and HC) have significantly diverse overall gut microbial flora, *P* < 0.05 (Figure 1(d)).

Comparatively, alpha diversity indices were nonsignificantly decreased in the NT subgroup compared to the HC subjects. Between the diabetic subgroups, all of Observed species, Chao1, and ACE significantly declined in the NT compared to MET and GLIM subgroups. Likewise, based on the Shannon index, the gut microbiome of the NT subgroup also showed a lower microbial diversity (Table 2).

### 3.3. Comparative Analysis of the Relative Abundance of Bacteria between HC and T2DM Subjects

**Figure 2:**
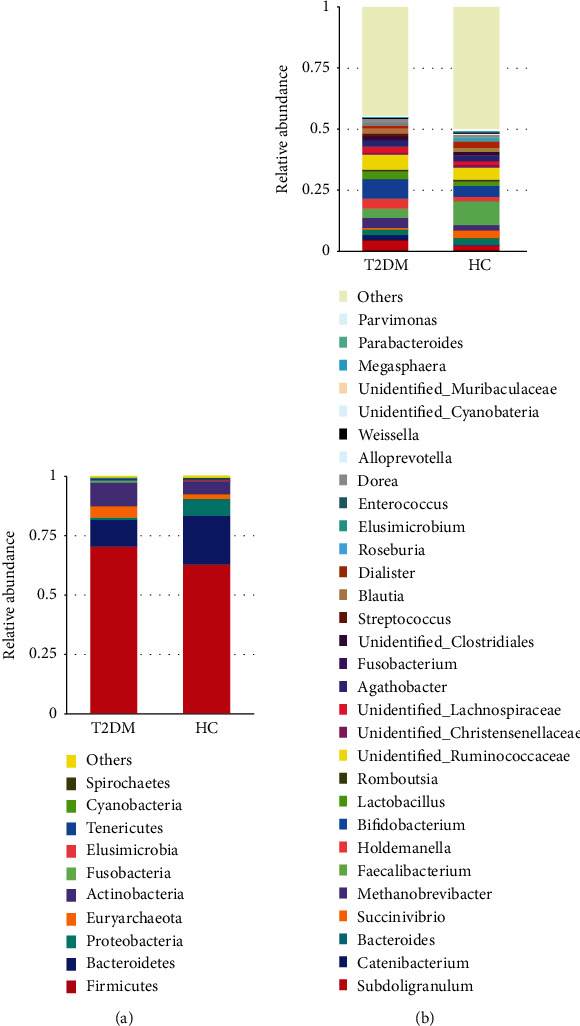
Comparison of gut microflora between HC (*n* = 24) and T2DM (*n* = 24) individuals. (a) and (b) show the relative abundance of bacteria at the phylum and genus level, respectively. HC: healthy control; *n*: number; T2DM: type 2 diabetes mellitus.

In either T2DM and HC group or diabetic subgroups, Firmicutes was the major abundant phylum. *Faecalibacterium* and *Bifidobacterium* were the major abundant genera in the HC and T2DM groups, respectively. At the level of relative abundant phylum and genera, there were numerous variations between the T2DM and HC groups (Figures 2(a) and 2(b); Table 3). These alterations were characterized by a significant rise in Actinobacteria and a decrease in Proteobacteria and Elusimicrobia phylum among T2DM individuals. In the genus level, a significant increase in the relative abundance of *Catenibacterium*, *Holdemanella*, *Bifidobacterium*, *Fusobacterium*, *Blautia*, and *Parvimonas* and reduction in *Succinivibrio*, *Faecalibacterium*, *Dialister*, and *Elusimicrobium* have also been observed among diabetic individuals compared to HC (Table 3). Likewise, apparent differences in the relative abundance of several taxa at both phylotype and genus levels have been detected between groups; however, it is not significant (Figures 2(a) and 2(b); Table 3). Similarly, the average of the Firmicutes-Bacteroidetes (F/B) ratio was nonsignificantly different between the T2DM (15.92) and HC groups (16.27), *P* = 0.117 (Table 2).

**Figure 3:**
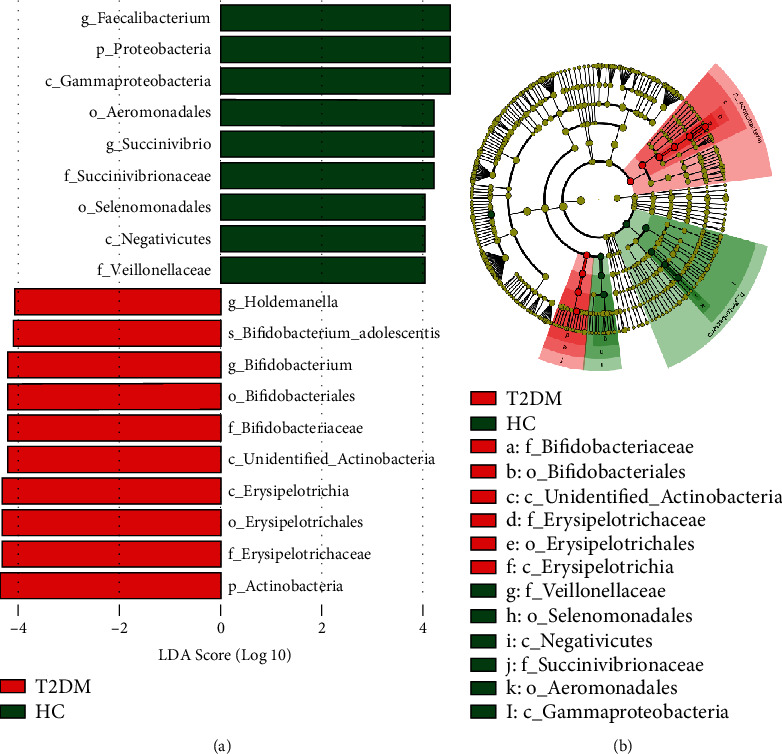
The LEfSe analysis findings. (a) The most deferentially abundant taxa in HC (*n* = 24) and T2DM (*n* = 24) individuals have been analyzed using LEfSe (LDA effect size software). Linear discriminant analysis (LDA) scores reveal the statistically significant biomarkers between the groups (biomarker with LDA score greater than the set value, the default setting is 4). (b) The taxonomy of the most deferentially abundant taxa. HC: healthy control; *n*: number; T2DM: type 2 diabetes mellitus.

Additionally, LEfSe analysis was further involved in identifying the bacterial taxa that exhibited significant differences between groups and exploring their taxonomy (Figures 3(a) and 3(b)). The major abundant bacterial taxa in diabetic individuals were Actinobacteria phylum, in particular, the members of Unidentified-Actinobacteria genera, and also the genus *Bifidobacterium* and it is higher taxonomy levels (order and family) as well as *Holdemanella* (Firmicutes) and it is upper classification stages starting from family to class. In contrast, of the major depleted bacterial taxa were the genus *Faecalibacterium* (Firmicutes) and *Succinivibrio* (Proteobacteria) (Figure 3(a)).

### 3.4. Effect of Antidiabetic Agents on Gut Microbiota of T2DM

Notably, *Methanobrevibacter* and *Bifidobacterium*, *Bifidobacterium* and Unidentified-Ruminococcaceae, and *Subdoligranulum* and *Bifidobacterium* were the major abundant genera in MET, GLIM, and NT subgroups, respectively (Table 3). There was no significant variation in F/B ratio between the NT and antidiabetic subgroups or the HC group; however, it is increased to 20.86 in the NT subgroup (Table 2). Moreover, a marked reduction of Proteobacteria (*P* < 0.05) and Elusimicrobia (*P* < 0.05) and an increase of Actinobacteria phylum (*P* < 0.05) have been detected in the NT subgroup compared to the HC group. At the genus level, the NT subgroup has also shown a lower abundance of several beneficial bacterial taxa including SCFA producers such as members of *Bacteroides*, *Faecalibacterium* (*P* < 0.05), *Dialister* (*P* < 0.05), *Fusobacterium* (*P* < 0.05), *Roseburia*, *Elusimicrobium* (*P* < 0.05), and *Megasphaera* compared to the healthy subjects (Table 3). Furthermore, antidiabetic agents restored to some extent the richness and diversity of gut microbiota (Table 2) and increased the relative abundance of many beneficial bacteria with a significant increase of *Methanobrevibacter* in MET compared to the NT subgroup (Table 3). Likewise, MET and GLIM enriched the gut of T2DM individuals with several beneficial microbial flora, including *Lactobacillus* and *Megasphaera*. In contrast, they have lowered the abundance of some opportunistic pathogens such as *Enterococcus* (Table 3).

### 3.5. Link of Gut Microbiota with T2DM, Age, and BMI

**Figure 4:**
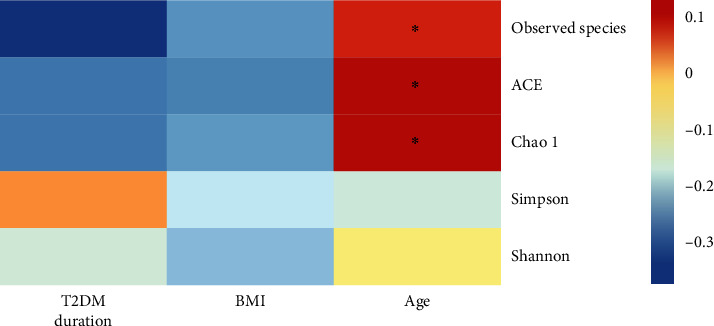
Correlation of alpha diversity indices with age (*n* = 48), BMI (*n* = 48), and diabetes duration (*n* = 24) of the study participants. BMI: body mass index; *n*: number; T2DM: type 2 diabetes mellitus.

Seemingly, the increase in diabetes duration resulted in the lowering OTUs of several samples and a reduction in gut microbiota richness and diversity as determined by a decline in the abundance of many bacteria (Table 4) as well as seen in ACE, Chao1, Shannon, and Observed species indices (Figure 4; SF 1). Furthermore, the correlation analysis found that many beneficial microbial taxa such as *Lactobacillus* and *Megasphaera* were negatively correlated with the diabetes duration. Likewise, several short-chain fatty acid producers have shown a negative correlation with FBG and HbA1c (Table 4). In contrast, *Bacteroides* was significantly positively correlated with FBG. Moreover, *Bifidobacterium*, *Lactobacillus*, Unidentified-Lachnospiraceae, and *Dorea* showed a significantly negatively correlation with HbA1c, whereas Unidentified-Ruminococcaceae and Unidentified-Cyanobacteria displayed a significantly positively correlation with HbA1c (Table 4).

**Figure 5:**
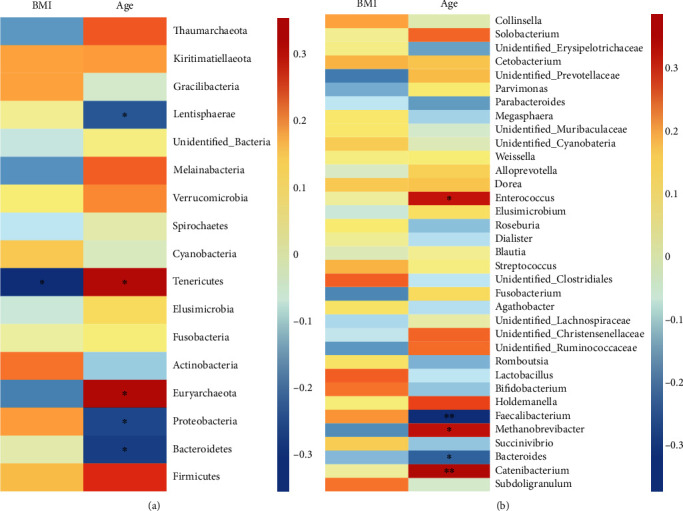
Correlation of age (*n* = 48) and BMI (*n* = 48) of the study participants with the relative abundance gut flora at both (a) phylum and (b) genus levels. ∗*P* < 0.05 and ∗∗*P* < 0.01. BMI: body mass index; *n*: number.

However, the microbial richness was positively correlated with age; the analysis based on Shannon index has shown that this augmentation accompanied by a nonsignificantly increased diversity in the overall commensal flora. Furthermore, with the increase in BMI, we detected an obvious decline in microbiota richness and diversity (Figure 4; SF 1). These shifts were also shown on the negative correlation of age and BMI (Figures 5(a) and 5(b); SF 2) with many beneficial microbiota such as the member of *Bacteroides* (*P* < 0.05), *Faecalibacterium* (*P* < 0.01), *Subdoligranulum*, *Bifidobacterium*, *Lactobacillus*, *Agathobacter*, *Dialister*, *Roseburia*, *Parabacteroides*, and *Megasphaera* with age as well as *Blautia*, *Parabacteroides*, and *Methanobrevibacter* with BMI (Figure 5(b); SF 2).

## 4. Discussion

Globally, diabetes mellitus is a persistent health challenge with inevitable complications [1, 2]. The exact mechanisms of T2DM development are not fully clear, though it is believed that the environmental elements play a critical role in the disease progression. Identifying the healthy ecosystem and intestinal microbial interaction is helpful to recognize the underlying mechanisms of disease and antidiabetic therapy. The current study revealed numerous changes in the gut microbial community of diabetic individuals and shift away from healthy subjects. These alterations were characterized by a decrease in the relative abundance of some SCFA producers such as *Faecalibacterium* and *Roseburia*, particularly in the NT subgroup compared to the HC group. Previous studies documented many changes in gut microbiota of T2DM individuals [20–23]. Some differences that existed between the studies may be explained by the variation in food habits, body weight, disease duration, and diabetic management policy and duration. Moreover, one of our key findings was divergent in the major abundant genera; however, the major abundant phylum among the study groups and subgroups was Firmicutes. This is consistent with previous literature [20, 24] that indicated Firmicutes as the major abundance phylum in healthy and T2DM individuals. Unlike our results, Salamon et al. study [20] found that the bacteria belong to unnamed genera in Ruminococcaceae were most dominant among both HC and T2DM individuals. In this study, we also observed a higher abundance of Firmicutes, *Lactobacillus*, and *Subdoligranulum* in the T2DM group compared to HC subjects, which are analogous to the findings of Ahmad et al. study that reported a higher abundance for these bacteria in obese-T2DM than HC individuals [24]. Likewise, in line with Salamon et al. study [20], *Bacteroides* and *Roseburia* were more in the HC subjects compared to the T2DM group, whereas *Blautia* and *Dorea* were more in the T2DM individuals compared to the HC group. Dissimilar to our study, Salamon et al. [20] and Ahmad et al. study [24] reported that the abundance of *Bifidobacterium* and *Dialister* was more in the HC subjects compared to the T2DM group and less in the HC subjects compared to the obese-T2DM group, respectively. Interestingly, the present study observed a decline in bacterial diversity (Based on Shannon) among T2DM subjects, in particular, in the individuals of the MET and NT subgroups compared to the HC group, which is in line with Zhang et al. [22] and Tao et al. study [23]. Likewise, the F/B ratio was higher in HC individuals compared to the T2DM group or the MET subgroup but lower compared to the NT subgroup. Previously, Salamon et al. study [20] reported a significantly higher F/B ratio in the T2DM group than in HC subjects. Furthermore, there was a gradual decline in gut microbiota richness and diversity with diabetes duration and the correlation analysis found that several beneficial bacteria were negatively correlated with the duration of illness, which indicates that the observed alterations are extremely dependent on disease duration. In general, our results are consistent with earlier studies [20–23], which confirmed the dysbiosis in T2DM individuals and added details about the link of T2DM with gut microbiota.

Interestingly, between the diabetic subgroups, many variations in gut microbial communities have appeared. Antidiabetic drugs not only improved the richness and diversity of gut microbes but also enriched the gut ecosystem with many beneficial microbes such as SCFA and vitamin producers, lowered the abundance of some opportunistic pathogens such as *Streptococcus* and *Enterococcus* genera, and restored to some extent the elevated F/B ratio. Formerly, many studies presented similar effects concerning MET [25–28]. In line with our study findings, Tong et al. study [26] found that MET significantly increased bacterial diversity and altered the gut microbiota structure of T2DM patients. Likewise, Zhang et al. study [22] reported a higher abundance of Spirochaetes in the MET group compared to NT subjects. Moreover, in agreement with de la Cuesta-Zuluaga et al. study [25], we found that the abundance of *Bifidobacterium* and *Megasphaera* was more in MET subcategory compared to HC group and NT subgroup, respectively. Unlike de la Cuesta-Zuluaga et al. study [25], *Megasphaera* was more in HC individuals compared to the MET subgroup. Taken together, these findings indicated that the gut microbiota of MET, GLIM, and NT subgroups were divergent as well as highlighted the relation of antidiabetic agents and gut microbiota, and suggested the protecting effect of antidiabetic agents against some opportunistic pathogens. In diabetic individuals, the weak immune system and low abundance of SCFA producers allow the increase of the opportunistic pathogens, which may later be associated with several complications. *Streptococcus* and *Enterococcus* have many members of opportunistic pathogens reported to cause intra- or extraintestinal diseases such as bacterial endocarditis, spontaneous bacterial peritonitis, and urinary tract infections [29–31].

Notably, *Bacteroides* was significantly positively correlated with FBG, whereas Fusobacteria showed a tendency for positive correlation, which is opposed to Yamaguchi et al. [32] and Tao et al. [23] study, respectively. The reason behind the dissimilarity between the studies may be the variation in nutritional habits and genetics, which are known factors that affect the gut microbiota [1, 2]. In the current study, *Bifidobacterium*, *Lactobacillus*, Unidentified-Lachnospiraceae, and *Dorea* have displayed a significant negative correlation with HbA1c; and several short-chain fatty acid producer bacteria have also shown a negative correlation with FBG and HbA1c; however, it is not significant. The study results are in line with studies [20, 23] that reported a significant negative correlation for *Faecalibacterium* with HbA1c. Previously, Tao et al. [23] study showed significant negative correlations for *Roseburia* with HbA1c that is analogous to the current study result. These findings underscored the link underlying the effects of gut microbiota imbalance in diabetic patients. Dysbiosis not only limited the role of intestinal microbiome but also increased the risk of diabetic complications. Extensive exposure to an unbalanced ecosystem can extremely disturb many pathways involved in insulin signaling and production. Modern studies showed that SCFAs improve blood glucose level and metabolic syndrome [33]. Indeed, SCFAs have several receptors interrelated to sugar homeostasis and insulin signaling. In the intestine, it is increasing the expression of peptide YY and GLP1, which enhance insulin secretion and reduce body weight through appetite regulation [7, 8]. Furthermore, it improves insulin signaling in the liver, adipose tissue, and muscle as well as enhances lipogenesis and oxidative metabolism, reduces the inflammatory reaction, and promotes energy expenditure [7, 8, 33–36]. Butyrate is also maintaining gut barrier function via the regulation of mucin 2 and tight-junction protein expressions, for example claudin-1 and Zonula Occludens-1, which prevent bacterial and lipopolysaccharide (LPS) translocation and systemic inflammation [8, 34]. Thus, the decline of SCFAs or, in particular, butyrate producer bacteria in type 2 diabetes individuals is directly affecting insulin signaling and sugar homeostasis. Moreover, however, the exact contribution quantity of B and K vitamins by gut microbial flora is unknown; dysbiosis of vitamin producers such as members of *Bifidobacterium* and *Lactobacillus* may extremely affect several aspects of life [37–39].

Regarding the relationship underlying the link of age and BMI with gut microbiota, the correlation analysis found that gut microbiota diversity and abundance of several beneficial bacteria were declined with BMI. We also found a significant negative correlation for *Faecalibacterium* and *Bacteroides* with age. In line with this study, Tao et al. [23] study reported that *Dialister* has a significant negative correlation with age, whereas in disagreement, they [23] found that *Roseburia* was significantly negatively correlated with BMI. Formerly, Salamon et al. [20] and Jandhyala et al. [40] studies found a significant negative correlation for the Ruminococcaceae genera and *Bacteroides* with BMI, respectively, which is similar to our study. Unlike our study, Salamon et al. study [20] found a significant negative correlation for *Streptococcus* with BMI. Analogous to Sedighi et al. study [41], there was a positive correlation between BMI and the quantity of *Lactobacillus.* Whereas, dissimilar to Sedighi et al. study [41], our study found that *Fusobacterium* was negatively correlated with BMI and *Bifidobacterium* displays a positive correlation with BMI, but it is not significant, which may be explained by the variation in dietary habits and lifestyle. Collectively, these findings highlighted the relationship of BMI and age with gut microbiota, and suggested the lack of potential roles of some beneficial microbes. In this regard, the link of bacteria with age and BMI requires further verification and investigations.

Although other studies are necessary to identify the microbiological alterations interrelated to T2DM, our findings are in the same line with recent studies and added a novel understanding about the pathogenesis of the disease, mechanisms underlying antidiabetic therapy, and the interaction of intestinal microbial flora with FBG, HbA1c, age, and BMI, which are of potential interest for further studies.

## 5. Conclusion

In summary, our study highlighted the particular alterations of gut microbiota in T2DM. Antidiabetic agents improve the richness and diversity of gut bacterial microbiota, enrich the gut ecosystem with beneficial microbes, and serve as a fighter against some opportunistic pathogens. Further studies should particularly focus on the interactions of gut microbiota with HbA1c, age, and BMI.

## Figures and Tables

**Table 1:** Characteristics of the study participants.

	Variable	HC (*n* = 24)	T2DM (*n* = 24)	*P* value	Diabetic clusters				*P* value for HC versus NT
					MET (*n* = 8)	GLIM (*n* = 8)	NT (*n* = 8)	*P* value	
Gender	Male	12	12		4	4	4		
	Female	12	12	1.000	4	4	4	1.000	1.000

Age (years)		47.12	53.87	0.024	57.37	51.25	53.00	0.366^b^	0.188

Marital status	Married	22	23		8	8	7		
	Single	2	1	1.000	0	0	1	1.000	1.000

Residence	Urban	19	15		4	4	7		
	Rural	5	9	0.341	4	4	1	0.261	1.000

Education level	No formal	5	5		2	3	0		
	Primary school	8	10	0.956	3	4	3	0.156	0.605
	Secondary school	6	5		3	0	2		
	University	5	4		0	1	3		

Occupation	Unemployed	11	12		6	4	2		
	Employee	6	6	1.000	0	1	5	0.060	0.225
	Free business	7	6		2	3	1		

BMI (kg/m^2^)		24.90	24.62	0.496^a^	24.51	23.76	25.58	0.854^c^	0.663

Blood glucose (mg/dl)	FBG	79.79	148.25	< 0.001^a^	154.87	118.75	171.12	0.531^b^	0.006^a^
	2 h	123.50	—	—	—	—	—	—	—

HbA1c (%)		—	6.15	—	6.12	5.81	6.51	0.044^c^	—

T2DM duration (years)		—	3.91	—	3.00	4.25	4.50	0.223^c^	—

For numerical data, the statistical analysis was conducted using two independent-samples *t*-test (parametric), Mann-Whitney *U* test^a^ (nonparametric), one-way ANOVA^b^ (parametric), and Kruskal-Wallis test^c^ (nonparametric). Fisher's exact test was involved for categorical data. Categorical and numerical data was presented as number and mean, respectively. A *P* value of less than 0.05 was considered significant. BMI: body mass index; FBG: fasting blood glucose; GLIM: glimepiride; HbA1c: hemoglobin A1c; HC: healthy control; MET: metformin; *n*: number; NT: nontherapeutic; T2DM: type 2 diabetes mellitus.

**Table 2:** Effect of T2DM and antidiabetic agents on alpha diversity indices and Firmicutes/Bacteroidetes ratio.

	Variable	HC (*n* = 24)	T2DM (*n* = 24)	*P* value	Diabetic clusters				*P* value for HC versus NT
					MET (*n* = 8)	GLIM (*n* = 8)	NT (*n* = 8)	*P* value	
Alpha diversity indices	Observed species	343.29	370.33	0.262	400.12	399.12	311.75	0.039^b^	0.337
	Chao1	400.79	457.65	0.054	498.27	486.74	387.96	0.045^b^	0.742
	ACE	404.97	456.43	0.058	494.79	481.64	392.84	0.049^b^	0.731
	Shannon	5.76	5.63	0.523^a^	5.49	6.09	5.29	0.050^c^	0.160^a^
	Simpson	0.95	0.94	0.369^a^	0.93	0.96	0.93	0.057^c^	0.220^a^

F/B ratio		16.27	15.92	0.117^a^	12.02	14.88	20.86	0.852^c^	0.380^a^

The statistical analysis was performed by two independent-samples *t*-test (parametric), Mann-Whitney *U* test^a^ (nonparametric), one-way ANOVA^b^ (parametric), and Kruskal-Wallis test^c^ (nonparametric). Data presented as mean. A *P* value of less than 0.05 was considered significant. B: Bacteroidetes; F: Firmicutes; GLIM: glimepiride; HC: healthy control; MET: metformin; *n*: number; NT: nontherapeutic; T2DM: type 2 diabetes mellitus.

**Table 3:** Difference of relative abundance phylum and genera between healthy and T2DM individuals and the influence of T2DM and antidiabetic agents on relative abundance microbiota.

	Gut microbiota	Study group		Diabetic subgroups		
		T2DM (*n* = 24)	HC (*n* = 24)	MET (*n* = 8)	GLIM (*n* = 8)	NT (*n* = 8)
Phylum	Firmicutes	0.70538	0.62941	0.64357	0.73542	0.73715
	Bacteroidetes	0.11494	0.20621	0.10678	0.10833	0.12972
	Proteobacteria	0.00819	0.06955∗∗	0.00540	0.00976	0.00941∗^h^
	Euryarchaeota	0.04487	0.02236	0.09897	0.03353	0.00211
	Actinobacteria	0.10489∗∗	0.05399	0.11575	0.09478	0.10415∗^h^
	Fusobacteria	0.00645	0.00144	0.01157	0.00020	0.00758
	Elusimicrobia	0.00002	0.00284∗	0.00005	0.00002	0.00000∗^h^
	Tenericutes	0.01000	0.01000	0.01000	0.01000	0.01000
	Cyanobacteria	0.00177	0.00011	0.00497	0.00008	0.00026
	Spirochaetes	0.00042	0.00389	0.00048∗^m^	0.00077	0.00002

Genera	Subdoligranulum	0.04409	0.02359	0.01118	0.02393	0.09716
	Catenibacterium	0.02132∗∗	0.00218	0.00391	0.00561	0.05444
	Bacteroides	0.02639	0.02676	0.05596	0.00781	0.01539
	Succinivibrio	0.00129	0.03231∗	0.00026	0.00282	0.00078
	Methanobrevibacter	0.04482	0.02224	0.09894∗^m^	0.03350	0.00202
	Faecalibacterium	0.03673	0.09919∗∗∗	0.01871∗∗^m^	0.05300	0.03849∗^h^
	Holdemanella	0.04082∗	0.01657	0.06683	0.02723	0.02839
	Bifidobacterium	0.08112∗	0.04477	0.09200	0.07046	0.08090
	Lactobacillus	0.03338	0.01825	0.03788	0.03943	0.02284
	Romboutsia	0.00463	0.00810	0.00604	0.00501	0.00284
	Unidentified-Ruminococcaceae	0.06293	0.04849	0.05558	0.06855	0.06467
	Unidentified-Christensenellaceae	0.00687	0.01057	0.01601	0.00389	0.00070
	Unidentified-Lachnospiraceae	0.02335	0.01694	0.02584	0.02489	0.01933
	Agathobacter	0.02619	0.02413	0.02003	0.02862	0.02992
	Fusobacterium	0.00383∗	0.00045	0.01135	0.00008	0.00005∗^h^
	Unidentified-Clostridiales	0.01594	0.01028	0.01742	0.01372	0.01669
	Streptococcus	0.00999	0.00525	0.01164	0.00766	0.01067
	Blautia	0.02246∗	0.01521	0.02067	0.02684	0.01988
	Dialister	0.01113	0.02689∗∗	0.00712	0.01812	0.00815∗^h^
	Roseburia	0.00817	0.01250	0.00726	0.00709	0.01015
	Elusimicrobium	0.00002	0.00284∗	0.00005	0.00002	0.00000∗^h^
	Enterococcus	0.00273	0.00025	0.00026	0.00038	0.00756
	Dorea	0.01363	0.00885	0.01380	0.01649	0.01006
	Alloprevotella	0.00206	0.00685	0.00281	0.00060∗^g^	0.00276
	Weissella	0.00243	0.00390	0.00098	0.00024	0.00606
	Unidentified-Cyanobacteria	0.00177	0.00011	0.00497	0.00008	0.00026
	Unidentified-Muribaculaceae	0.00001	0.00157	0.00003	0.00000	0.00000
	Megasphaera	0.00095	0.00271	0.00091	0.00123	0.00070
	Parabacteroides	0.00258	0.00531	0.00197	0.00114	0.00462
	Parvimonas	0.00148∗∗	0.00000	0.00434	0.00006	0.00003

At 95% level of significance, Mann-Whitney *U* test (nonparametric data) and 2 independent-samples *t*-test^a^ (parametric data) assessed the difference between groups. Data expressed as mean. A *P* value of less than 0.05 was considered significant. ∗*P* < 0.05, ∗∗*P* < 0.01, and ∗∗∗*P* < 0.001. The significant *P* values were labeled by m, g, and h for the nontherapeutic subgroup (NT) versus metformin (MET), glimepiride (GLIM), and healthy control (HC), respectively. *n*: number; T2DM: type 2 diabetes mellitus.

**Table 4:** Link of relative abundance microbiota and T2DM.

	Gut microbiota	Duration of T2DM		Spearman correlation		
		1-5 years (*n* = 20)	6-10 years (*n* = 4)	Duration	FBG	HbA1c
Phylum	Firmicutes	0.68311	0.81671	0.062	-0.257	0.272
	Bacteroidetes	0.12843	0.04749	0.072	0.250	-0.277
	Proteobacteria	0.00714	0.01345	0.164	0.301	-0.183
	Euryarchaeota	0.05367	0.00086	-0.454∗	-0.120	0.199
	Actinobacteria	0.10364	0.11115	0.073	0.023	-0.412∗
	Fusobacteria	0.00773∗	0.00004	-0.414∗	0.074	-0.156
	Elusimicrobia	0.00003	0.00000	-0.166	0.047	0.009
	Tenericutes	0.01000	0.01000	0.059	0.105	0.229
	Cyanobacteria	0.00212	0.00004	-0.364	-0.270	0.418∗
	Spirochaetes	0.00048	0.00013	-0.316	0.089	0.031

Genera	Subdoligranulum	0.01783	0.17537	0.064	-0.324	-0.108
	Catenibacterium	0.00325	0.11168	0.064	-0.162	-0.342
	Bacteroides	0.03069	0.00489	0.351	0.446∗	0.192
	Succinivibrio	0.00094	0.00301	0.067	0.024	-0.315
	Methanobrevibacter	0.05364	0.00071	-0.466∗	-0.127	0.175
	Faecalibacterium	0.03720	0.03441	0.092	-0.870	-0.344
	Holdemanella	0.03348	0.07751	-0.051	-0.217	0.006
	Bifidobacterium	0.08066	0.08343	0.055	-0.038	-0.443∗
	Lactobacillus	0.03249	0.03787	-0.086	-0.158	-0.464∗
	Romboutsia	0.00546	0.00049	-0.190	0.203	0.262
	Unidentified-Ruminococcaceae	0.06656	0.04480	-0.197	-0.043	0.432∗
	Unidentified-Christensenellaceae	0.00821∗	0.00016	-0.306	-0.015	0.086
	Unidentified-Lachnospiraceae	0.02428	0.01871	0.000	-0.227	-0.486∗
	Agathobacter	0.02307	0.04179	0.125	-0.251	-0.320
	Fusobacterium	0.00459	0.00002	0.132	0.376	-0.144
	Unidentified-Clostridiales	0.01769	0.00721	-0.235	-0.037	0.112
	Streptococcus	0.01119	0.00401	-0.110	-0.083	-0.282
	Blautia	0.01937	0.03794	0.211	-0.020	-0.257
	Dialister	0.01247	0.00442	-0.140	-0.108	-0.301
	Roseburia	0.00852	0.00638	0.050	0.027	-0.199
	Elusimicrobium	0.00003	0.00000	-0.166	0.047	0.009
	Enterococcus	0.00026	0.01512	-0.082	-0.125	0.174
	Dorea	0.01137	0.02493	0.094	-0.352	-0.430∗
	Alloprevotella	0.00232	0.00071	-0.492∗	-0.020	0.195
	Weissella	0.00281	0.00056	-0.185	0.180	0.030
	Unidentified-Cyanobacteria	0.00212	0.00004	-0.364	-0.270	0.418∗
	Unidentified-Muribaculaceae	0.00001	0.00000	-0.018	0.303	-0.002
	Megasphaera	0.00094	0.00098	-0.255	-0.146	0.114
	Parabacteroides	0.00246	0.00316	0.130	0.278	0.276
	Parvimonas	0.00177	0.00001	-0.115	0.101	-0.239

The statistical difference between groups was assessed by Mann-Whitney *U* test (nonparametric data) and 2 independent-samples *t*-test^a^ (parametric data). Data presented as mean. A *P* value of less than 0.05 was considered significant. ∗*P* < 0.05. FBG: fasting blood glucose; HbA1c: hemoglobin A1c; *n*: number; T2DM: type 2 diabetes mellitus.

## Data Availability

The sequence data used in this study are available in NCBI (Accession number PRJNA588353).

## Supplementary Materials

Supplementary 1 Supplementary file 1: Correlation of age, BMI, and T2DM duration with alpha diversity indices.

Supplementary 2 Supplementary file 2: Correlation of age and BMI with relatively abundant bacteria at both the phylum and genus levels.
